# Unveiling novel biomarkers for platinum chemoresistance in ovarian cancer

**DOI:** 10.1515/med-2024-1084

**Published:** 2025-01-13

**Authors:** Caixia Wang, Changsheng Peng, Chuan Xie

**Affiliations:** Department of Obstetrics and Gynecology, West China Second University Hospital, Sichuan University, Chengdu, Sichuan, P.R. China; Key Laboratory of Birth Defects and Related Diseases of Women and Children (Sichuan University), Ministry of Education, Chengdu, Sichuan, China

**Keywords:** platinum resistance, MFAP4, EFEMP1, epithelial–mesenchymal transition, immune infiltration, ovarian cancer

## Abstract

Primary chemoresistance to platinum-based treatment is observed in approximately 33% of individuals diagnosed with ovarian cancer; however, conventional clinical markers exhibit limited predictive value for chemoresistance. This study aimed to discover new genetic markers that can predict primary resistance to platinum-based chemotherapy. Through the analysis of three GEO datasets (GSE114206, GSE51373, and GSE63885) utilizing bioinformatics methodologies, we identified two specific genes, MFAP4 and EFEMP1. The findings revealed that the areas under the receiver operating characteristic curves for MFAP4 and EFEMP1 were 0.716 and 0.657 in the training cohort, and 0.629 and 0.746 in the testing cohort, respectively. In all cases or in cases treated with platin, high expression of MFAP4 and EFEMP1 was linked to shortened overall survival and progression-free survival. MFAP4 and EFEMP1 were positively correlated with epithelial–mesenchymal transition, TGF-β signaling, KRAS signaling, and so on. The high expression groups of MFAP4 and EFEMP1 exhibited elevated stromal, immune, and ESTIMATE scores. Finally, we constructed a regulatory network involving lncRNA–miRNA–mRNA interactions. In summary, MFAP4 and EFEMP1 have the potential to serve as predictive indicators for both response to platinum-based chemotherapy and survival rates, and might be regarded as innovative biomarkers and therapeutic targets for OC patients.

## 1 Introduction

Ovarian cancer (OC) is the most fatal form of gynecological cancer. According to the American Cancer Society, there will be 19,680 new cases and 12,740 deaths due to ovarian malignancy in the United States by 2024 [1]. The primary approach for managing OC involves tumor reduction surgery and initial chemotherapy, with carboplatin and paclitaxel being the preferred first-line treatments [2,3]. However, the development of chemotherapy resistance, particularly platinum resistance, significantly affects the prognosis of OC patients [4]. The initial response rates to first-line chemotherapy in individuals diagnosed with OC are limited to a range of 60–80%, and a portion of these individuals eventually acquire resistance to the drugs, resulting in an approximate 30% survival rate after 5 years [4,5]. Therefore, it is imperative to investigate the molecular mechanisms underlying the development of innovative strategies to overcome resistance.

Platinum-based medications belong to a category of broad-spectrum anticancer drugs that disrupt the structure and functionality of DNA within tumor cells, thereby exhibiting anticancer properties [6]. Platinum resistance refers to the recurrence of patients within 6 months of treatment with platinum drugs [4]. Drug metabolism, driver mutations, and tumor cell metabolism are closely associated with drug resistance in OC [7–9]. In addition, other factors, such as signal pathway changes and exosomes are also involved [10]. Activation of the focal adhesion kinase (FAK) signaling pathway has been shown to be associated with chemotherapy resistance. However, the combination of a FAK inhibitor with platinum can overcome chemoresistance and trigger apoptosis [11]. Heat-shock protein 90 has been identified by proteomic methods as a drug target for reversing platinum resistance in OC [12]. Repressing FOXM1 using thiostrepton results in a reduction of FOXM1 mRNA expression and its downstream effectors, such as CCNB1 and CDC25B, ultimately inducing cell death in OC [13]. Significant advancements have been made in elucidating the underlying mechanisms of chemoresistance in OC; however, numerous challenges remain to be addressed.

In this study, we employed weighted gene co-expression network analysis (WGCNA) along with various machine-learning methods to successfully identify and characterize two pivotal genes, MFAP4 and EFEMP1 that exhibit significant associations with chemotherapy resistance in OC. Subsequently, we conducted an extensive analysis using a comprehensive array of algorithms including gene set variation analysis (GSVA), survival analysis, and copy number alterations (CNAs). Furthermore, we investigated the immune correlation of these genes and constructed an lncRNA–miRNA–mRNA network. The findings presented herein have the potential to significantly contribute to the advancement of more efficacious and precisely targeted therapeutic interventions for OC.

## 2 Materials and methods

### 2.1 GEO data download and preprocessing

The GEO (http://www.ncbi.nlm.nih.gov/geo/) is a publicly available functional genomic data repository. To screen datasets related to platinum resistance, the gene expression profiles of GSE114206, GSE51373, and GSE63885 were downloaded from the GEO database. The three GEO series were merged and normalized using the R packages sva and limma, followed by visualization through principal component analysis (PCA). The limma package was employed to conduct variance analysis, with the filter condition set as |logFC| > 1.5 and *p* < 0.05.

### 2.2 WGCNA

WGCNA identifies modules of genes with similar expression patterns to explore potential correlations between genomes and clinical characteristics [14]. To conduct an unsigned WGCNA analysis, we used the WGCNA package (with parameters set as Soft-power 3, mergeCutheight 0.25, and minModuleSize 30). Genes were categorized based on their expression patterns, using the weighted correlation coefficients. Module membership (MM) refers to the correlation between module eigengenes and gene expression profiles. Finally, eight nongray modules were identified using WGCNA, and further investigation focused on 554 genes within the blue module.

### 2.3 Machine learning

To accurately predict the key genes involved in chemotherapy resistance, we utilized various methods including LASSO regression, support vector machine recursive feature elimination (SVM-RFE), and random forest (RF). These techniques allowed us to rank the importance of features using R packages such as “glmnet,” “e1071,” “kernlab,” “caret,” and “randomForest” [15–17]. The most relevant and feasible characteristics of the resistant subtype were confirmed as genes that converged using these three machine learning methods for feature selection.

### 2.4 Receiver operating characteristic (ROC) curves analysis

Each candidate hub gene was subjected to ROC curve analysis using the “pROC” package to verify its accuracy [18]. The results indicated that genes with an area under the curve exceeding 0.60 could potentially offer diagnostic advantages for illnesses in both the training and testing groups.

### 2.5 cBioPortal database analysis

The cBioPortal database (https://www.cbioportal.org) is an online platform that facilitates the exploration of DNA copy numbers, DNA methylation patterns, mRNA and microRNA expression levels, and non-synonymous mutations [19]. By leveraging this comprehensive resource, investigation into CNAs in OC can be conducted, specifically targeting the MFAP4 and EFEMP1 genes.

### 2.6 GSVA

GSVA is a statistical method employed to identify differentially expressed genes (DEGs) and gene sets within a given sample set, thereby offering valuable insights into the underlying biological processes and pathways associated with observed variation [20]. Subgroups were stratified based on the median gene expression values. The gene set “h.all.v7.5.1.symbols.gmt” was obtained from the MSigDB database. Differential analysis of the HALLMARK pathways was conducted using the R package “limma.” Enriched pathways were considered significant if they exhibited a *t*-value greater than 2 and *p*-value less than 0.05.

### 2.7 GeneMANIA analysis

The Genemania database (http://www.genemania.org) serves as a versatile resource for constructing protein–protein interaction networks, facilitating the visualization of functional connections between genes, and enabling comprehensive analysis of gene interactions and functions [21]. In this study, GeneMANIA was utilized to generate a core gene network to elucidate the underlying mechanism of action in patients with OC.

### 2.8 Immunocyte infiltration

The ESTIMATE algorithm was used with the assistance of the “estimate” package to calculate the stromal, immune, and ESTIMATE scores for each sample [22]. The relative abundance of 22 immune-related cell types within a diverse cell population was assessed using CIBERSORT, an analytical tool [23]. In this study, we investigated the correlation between gene expression levels and infiltrating immune cells, and the results are presented as lollipop plots.

IMPACT (http://www.brimpact.cn/) is an online platform that facilitates the investigation of predictive biomarkers for immunotherapy and cancer prognosis by utilizing genomic, transcriptomic, and proteomic data [24]. In this study, we explored the relationship between key genes associated with tumor purity and immune-related pathways.

### 2.9 Single cell analysis

TISCH (http://tisch.comp-genomics.org/) is an innovative database that offers comprehensive cell-type annotation at the single-cell level, enabling researchers to explore the complex tumor microenvironment (TME) across a wide range of cancer categories [25]. In this in-depth study, we focused on analyzing the expression of key genes in various cell types within the OV_GSE151214 dataset.

### 2.10 Competing endogenous RNA (ceRNA) network

miRNA target genes were predicted using TargetScan, miRanda, miRWalk, and miRDB. SpongeScan (http://spongescan.rc.ufl.edu/) was used to retrieve lncRNAs targeted by miRNAs. Cytoscape is a highly effective software tool for visualizing and analyzing network data, enabling the construction of intricate biological networks [26]. In the network diagram created by Cytoscape, nodes and edges are fundamental components. To construct a ceRNA network (lncRNA–miRNA–mRNA), we employed Cytoscape 3.7.1 as well.

### 2.11 Statistical analysis

Statistical analyses were performed using the R software version 4.2.1. Adobe Illustrator 2024 was used to generate the figures. Clinical survival analyses were conducted using the KMplotter (https://kmplot.com/analysis/). Statistical significance was determined based on a *p*-value <0.05.

## 3 Results

### 3.1 Identification of platinum-based chemoresistance related geneset

**Figure 1:**
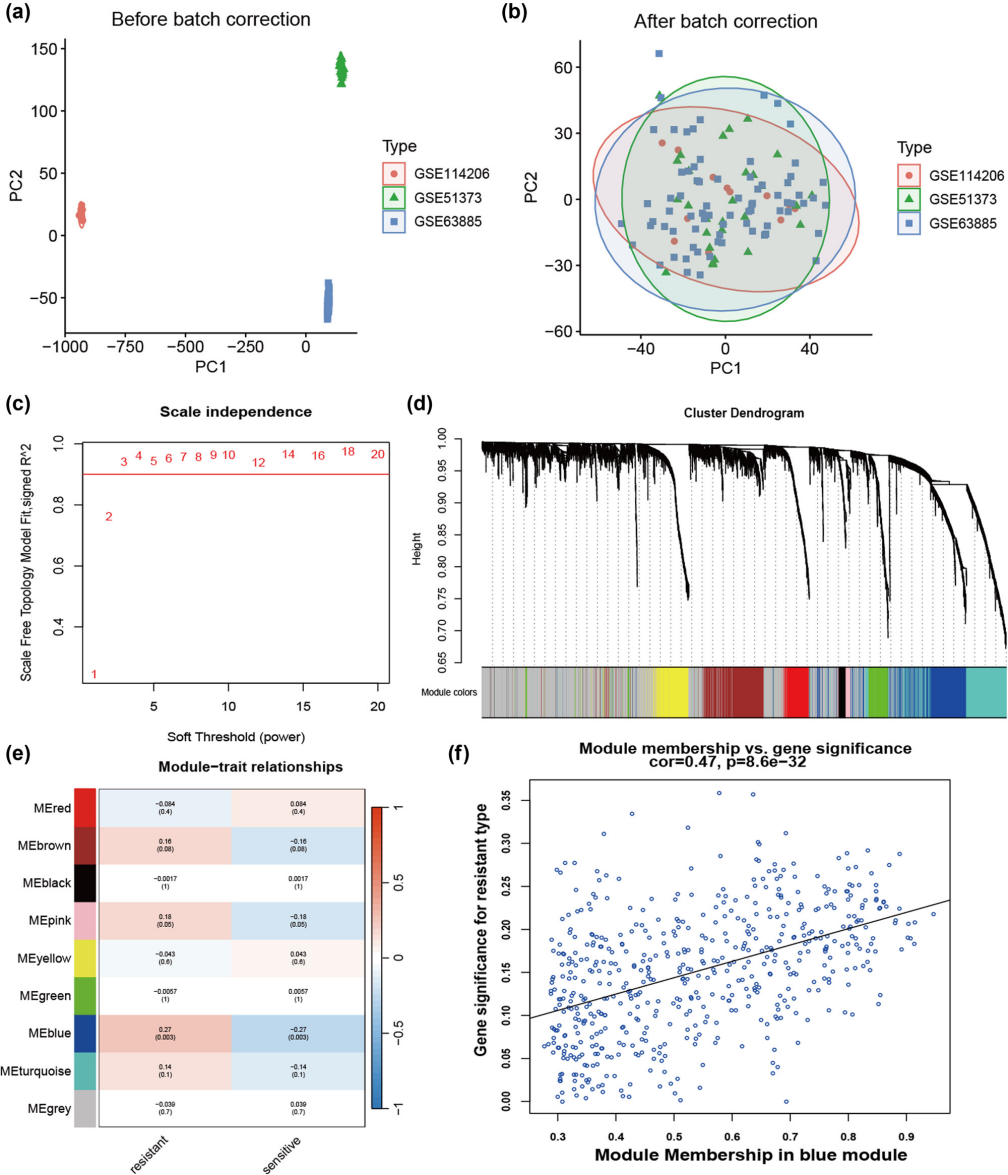
Identification of the hub module using WGCNA. (a) Three GEO datasets are merged and PCA plot before batch correction. (b) PCA plot after batch correction. (c) Analysis of the scale-free fit index of different soft threshold powers. (d) Clustering dendrogram of genes. (e) Correlation of these modules between the resistant group and sensitive group. (f) Scatter plot of the relationship between the blue module and the resistant group.

**Figure A1:**
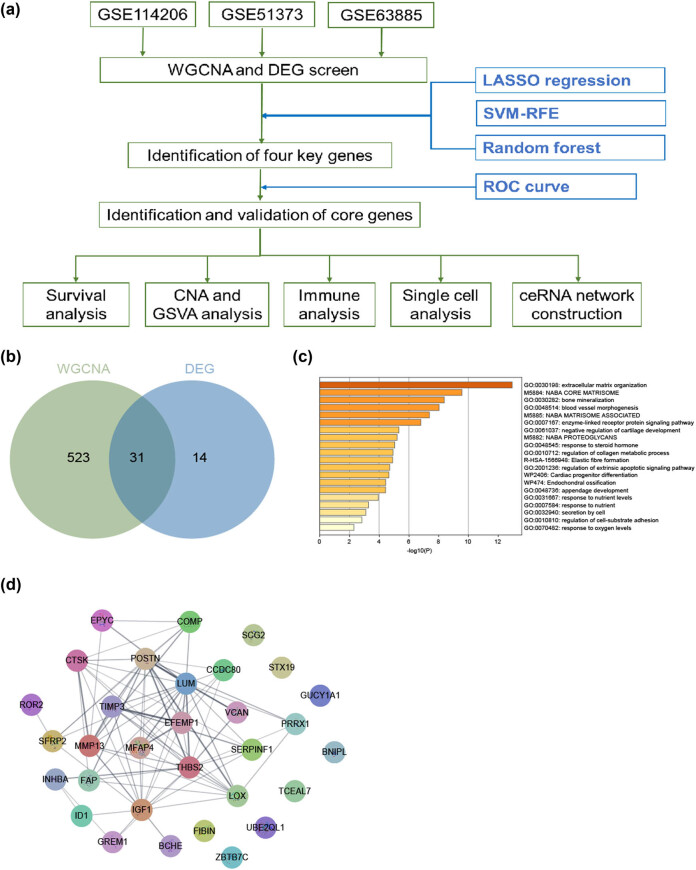
The exploration of intersect genes. (a) The flowchart of whole project. (b) The Venn diagram shows the common genes in both WGCNA and DEGs. (c) Enrichment analysis based on the Metascape database. (d) Construction of protein–protein interaction network using the string database.

The program flowchart for this study is presented in Figure A1a. To acquire a geneset associated with chemoresistance to platinum-based agents, we extracted RNA level profiles from three datasets: GSE114206 (6 samples of platinum-sensitive and 6 samples of resistant), GSE51373 (16 samples of platinum-sensitive and 12 samples of resistant), and GSE63885 (41 samples of platinum-sensitive and 34 samples of resistant). These datasets were merged and normalized for subsequent analyses (Figure 1a and b). Next, we employed WGCNA to construct a gene co-expression network and used *β* = 3 to establish a scale-free network (Figure 1c). Subsequently, a hierarchical clustering tree was constructed using dynamic hybrid cutting to identify gene modules. The branches of the tree revealed genes with comparable expression patterns (Figure 1d). Furthermore, eight non-gray modules were constructed and the blue module was identified as the candidate hub module (MM = 0.47, gene significance = 8.6 × 10^−32^) (Figure 1e and f).

### 3.2 Identification of key genes and enrichment analysis

**Figure 2:**
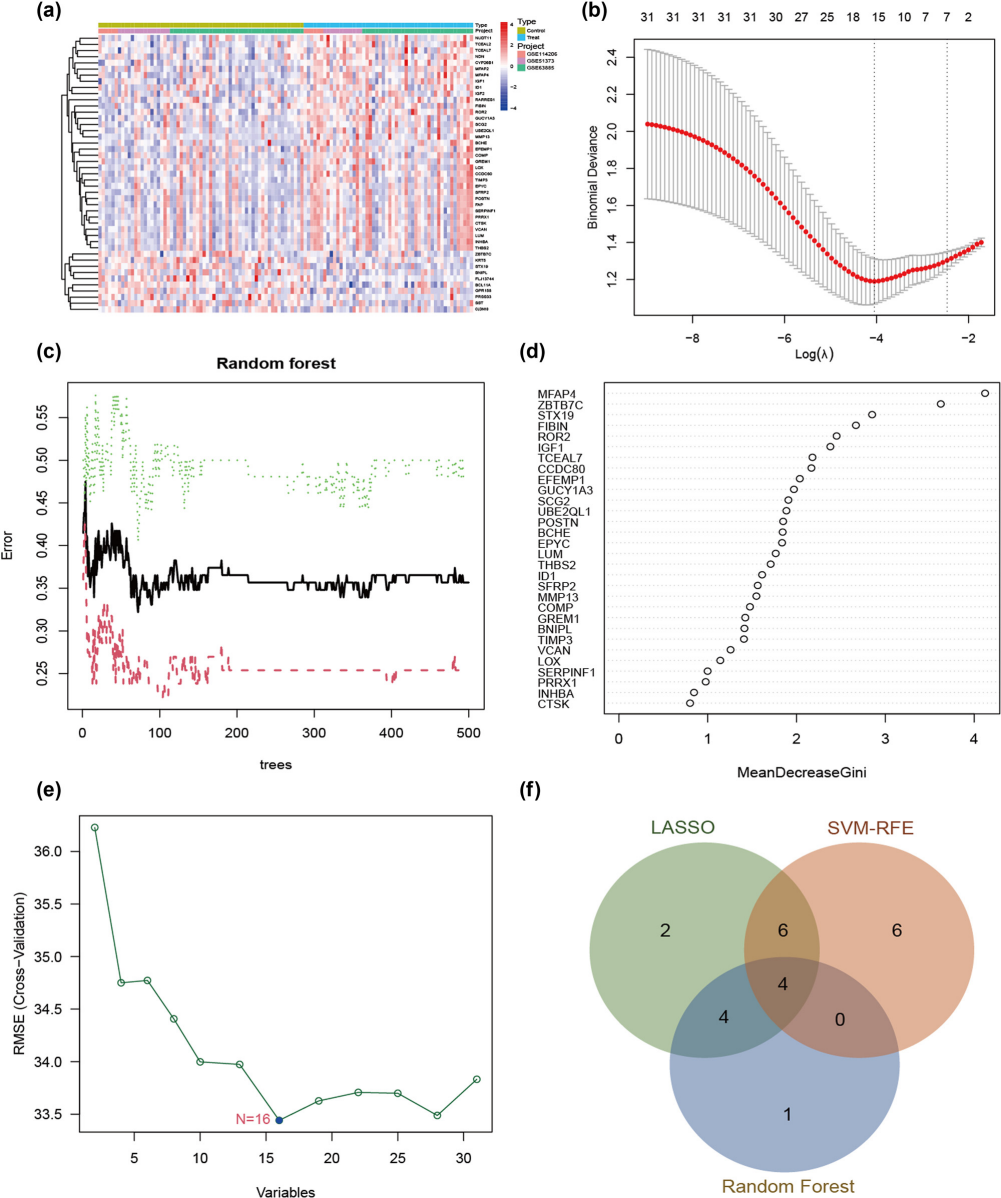
Screening for core genes in platinum-resistant group. (a) DEGs are shown on the heatmap. (b) Selection of the best Log (*λ*) value for LASSO regression. (c) Influence of the number of decision trees on the error rate. (d) Mean decrease Gini method in random forest classifier. (e) Variation curve of gene cross-validation error in SVM-RFE algorithm. (f) The Venn diagram shows the platinum-resistant genes shared by LASSO, SVM-RFE, and RF algorithms.

We screened 45 DEGs between the sensitive and resistant groups (|logFC| > 1.5, *p* < 0.05) (Figure 2a). Through the intersection of the DEG and WGCNA results, we identified 31 genes that were consistently identified in both analyses (Figure A1b). GO annotation using Metascape (https://metascape.org/) suggested that these genes are associated with epithelial–mesenchymal transition (EMT). In addition, we used STRING (https://string-db.org/) to construct a protein interaction network (Figure A1c and d).

To determine the core genes associated with chemoresistance in OC, we employed the LASSO regression, SVM-RFE, and RF algorithms for feature selection. LASSO regression analysis identified 16 variables, namely ROR2, MFAP4, GUCY1A3, FIBIN, IGF1, UBE2QL1, MMP13, STX19, EFEMP1, ZBTB7C, BNIPL CCDC80 ID1 THBS2 GREM1, and VCAN as crucial indicators associated with resistance to chemotherapy (Figure 2b). Nine genes, MFAP4, ZBTB7C, STX19, FIBIN, ROR2, IGF1, TCEAL7, CCDC80, and EFEMP1, were identified as signature genes with relative importance scores greater than two (Figure 2c and d). For the SVM-RFE algorithm, the error was minimized when the number of features was 16, including BCHE, EPYC, UBE2QL1, GREM1, SFRP2, MFAP4, MMP13, SCG2, POSTN, VCAN, ROR2, GUCY1A3, EFEMP1, ZBTB7C, BNIPL, and TIMP3 (Figure 2e). After this intersection, four common signature genes, ROR2, MFAP4, EFEMP1, and ZBTB7C, were identified (Figure 2f).

**Figure 3:**
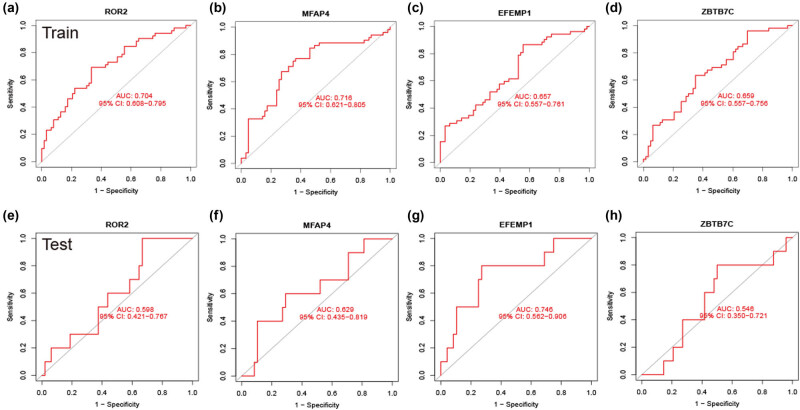
Evaluation of the diagnosis of core genes. (a)–(d) ROC curves of ROR2, MFAP4, EFEMP1, and ZBTB7C in the training group. (e)–(h) ROC curves of ROR2, MFAP4, EFEMP1, and ZBTB7C in the testing group (GSE30161). AUC value is the area under the ROC curve.

Candidate hub genes were identified using ROC curve analysis to ensure their accuracy. In the merged group, the area under the ROC curves (AUCs) for ROR2, MFAP4, EFEMP1, and ZBTB7C were 0.704, 0.716, 0.657, and 0.659, respectively (Figure 3a–d). In the external validation set GSE30161, the AUCs for these markers were 0.598, 0.629, 0.746, and 0.546, respectively (Figure 3e–h). Ultimately, MFAP4 and EFEMP1 were identified as potential markers for predicting platinum resistance in OC.

### 3.3 Survival analysis for key genes

**Figure 4:**
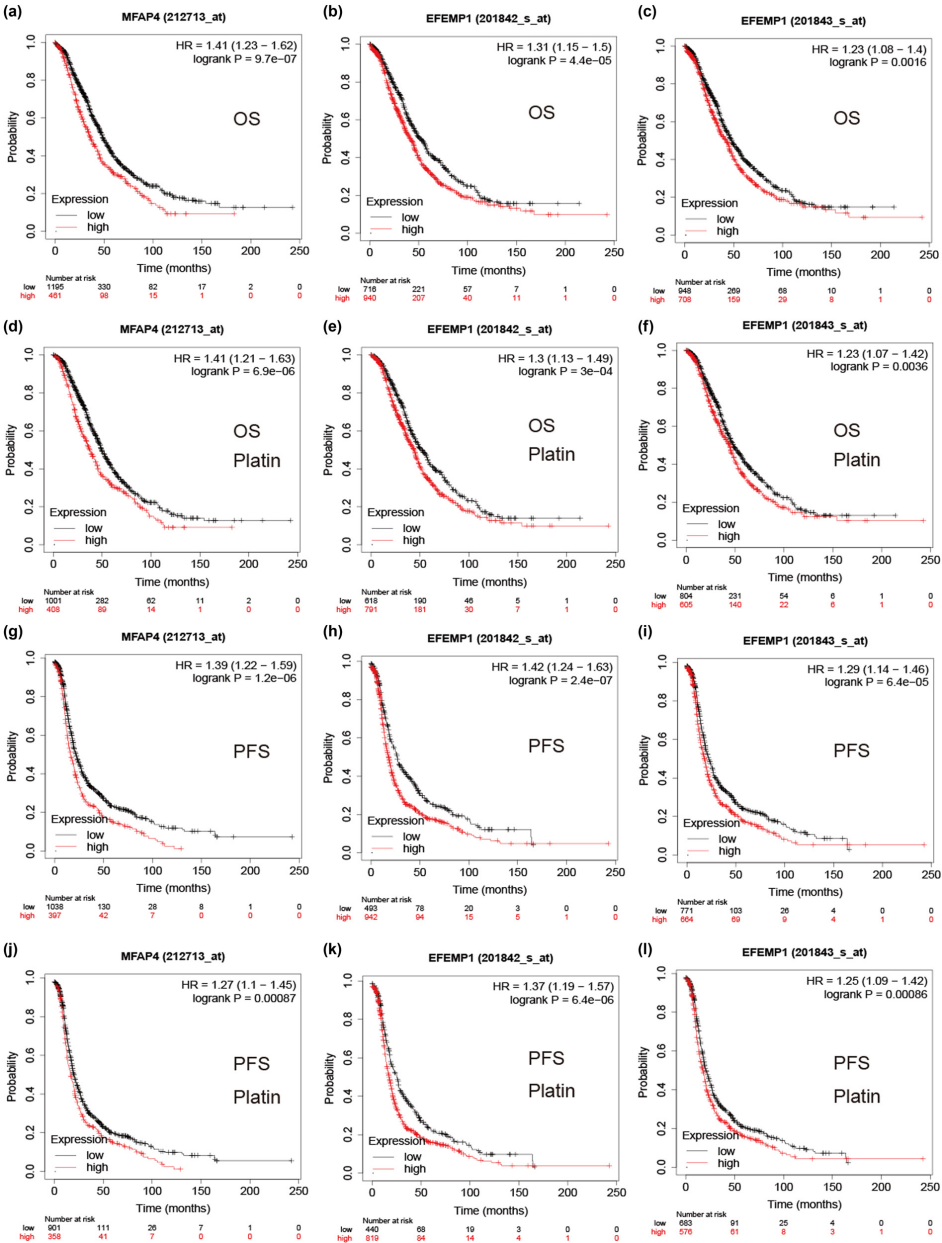
Relevance of core gene expression to the prognosis of patients with OC. (a)–(c) Relationship between MFAP4 and EFEMP1 expression and OS in patients with OC. (d)–(f) Relationship between MFAP4 and EFEMP1 expression and OS in patients with OC treated with platinum chemotherapy. (g)–(i) Relationship between MFAP4 and EFEMP1 expression and PFS in patients with OC. (j)–(l) Relationship between MFAP4 and EFEMP1 expression and PFS in patients with OC treated with platinum chemotherapy.

To investigate the association between MFAP4 and EFEMP1 expression and OC prognosis, we conducted a survival analysis using the Kaplan–Meier plotter database. In all cases or platinum-based chemotherapy cases, the findings demonstrated that high expression of MFAP4 and EFEMP1 was associated with shorter overall survival (OS) (Figure 4a–f), which was also observed in the progression-free survival (PFS) group (Figure 4j–l). These findings suggest a strong correlation between MFAP4 and EFEMP1 expression and platinum resistance and an unfavorable prognosis in OC.

### 3.4 Multiomics validation of core genes in OC

**Figure 5:**
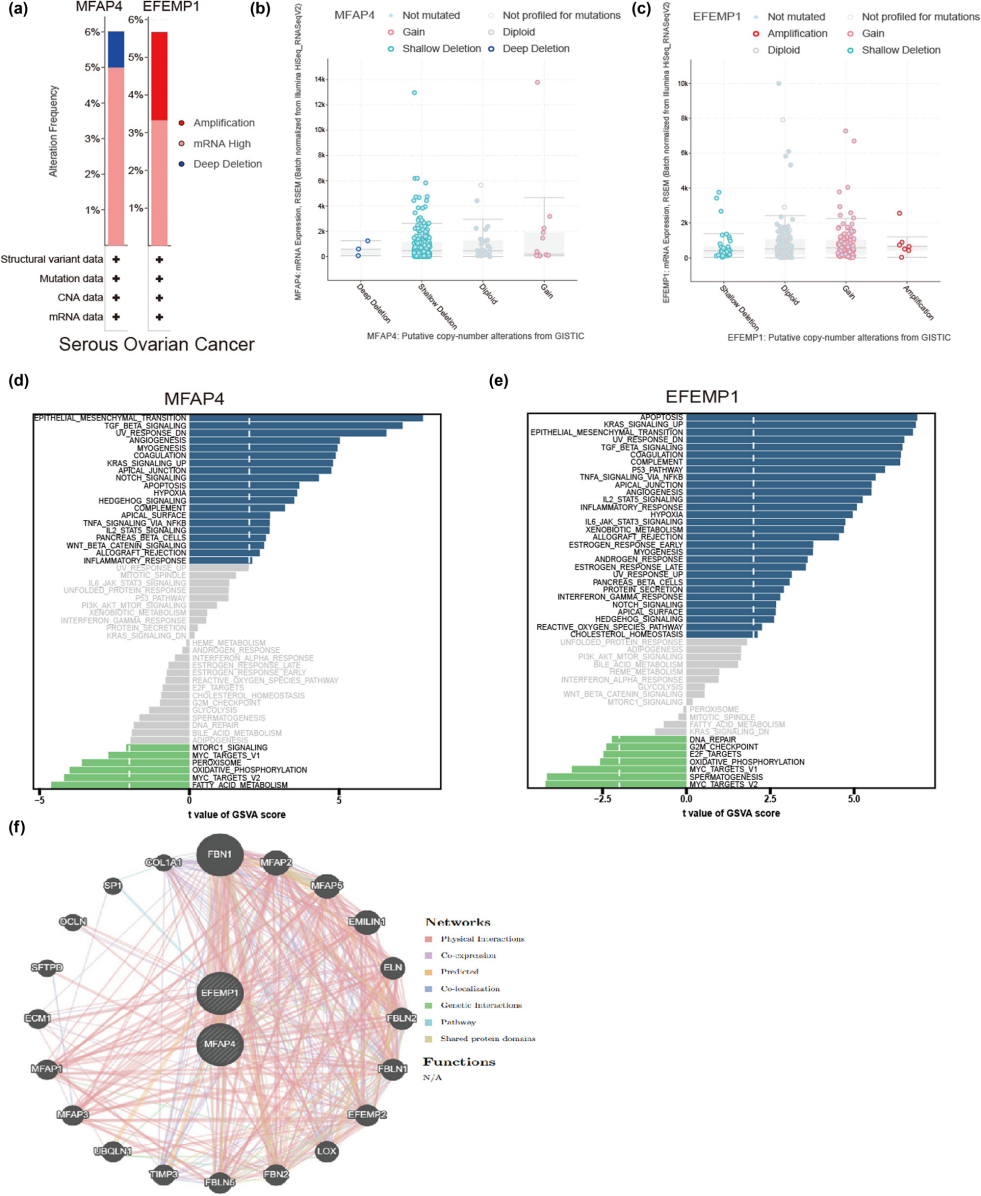
Multiomics of core genes. (a) Alteration frequency of MFAP4 and EFEMP1 in OC. (b) and (c) Putative CNAs of MFAP4 and EFEMP1. (d) and (e) GSVA of MFAP4 and EFEMP1. (f) Networks of MFAP4 and EFEMP1 based on the GeneMANIA database.

We examined the correlation between the expression of core genes and mutations, as well as CNA, using cBioPortal. As depicted in Figure 5a–c, critical factors associated with mutations were identified through gain and deep deletion events involving the MFAP4 gene, while amplification and gain events affecting the EFEMP1 gene were also found to significantly contribute to mutational processes. GSVA analysis showed that MFAP4 and EFEMP1 were positively correlated with the EMT, TGF-β signaling, KRAS signaling, and so on (Figure 5d and e). The MFAP4 and EFEMP1 networks were visualized using GeneMANIA (Figure 5f).

### 3.5 Relationship between core genes and immune infiltration in OC

**Figure 6:**
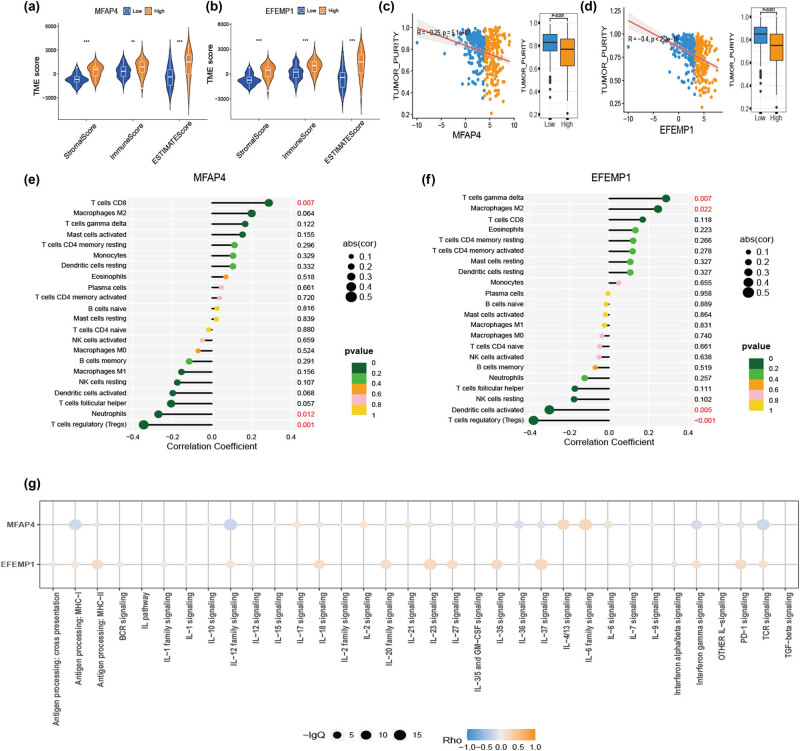
Correlation of core genes and TME. (a) and (b) Differences in the immune score, ESTIMATE score, and stromal score were observed in the expression of MFAP4 and EFEMP1. (c) and (d) Differential tumor purity in the expression profiles of MFAP4 and EFEMP1. (e) and (f) Correlation analysis between expression of MFAP4 and EFEMP1 and immune cells. (g) Immune-related pathways of MFAP4 and EFEMP1 using the IMPACT database.

To investigate the association between TME characteristics and the two genes, our findings indicated that elevated stromal, immune, and ESTIMATE scores were observed in cases with high expression of MFAP4 and EFEMP1 (Figure 6a and b). Additionally, a negative correlation was found between high expression of MFAP4 and EFEMP1 and tumor purity (Figure 6c and d). Furthermore, using the CIBERSORT method to evaluate the relationship between these two genes and 22 types of immune cells revealed a positive correlation between high expression of MFAP4 and T cell CD8 levels (Figure 6e), as well as a positive correlation between high expression of EFEMP1 and T cell gamma delta levels along macrophages M2 (Figure 6f). Moreover, an analysis focusing on immune-related pathways associated with MFAP4 and EFEMP1 genes demonstrated increased IL-6 family signaling for MFAP4 and increased IL-37 signaling for EFEMP1 (Figure 6g).

### 3.6 Analysis of TME-associated cells and construction of ceRNA network

**Figure 7:**
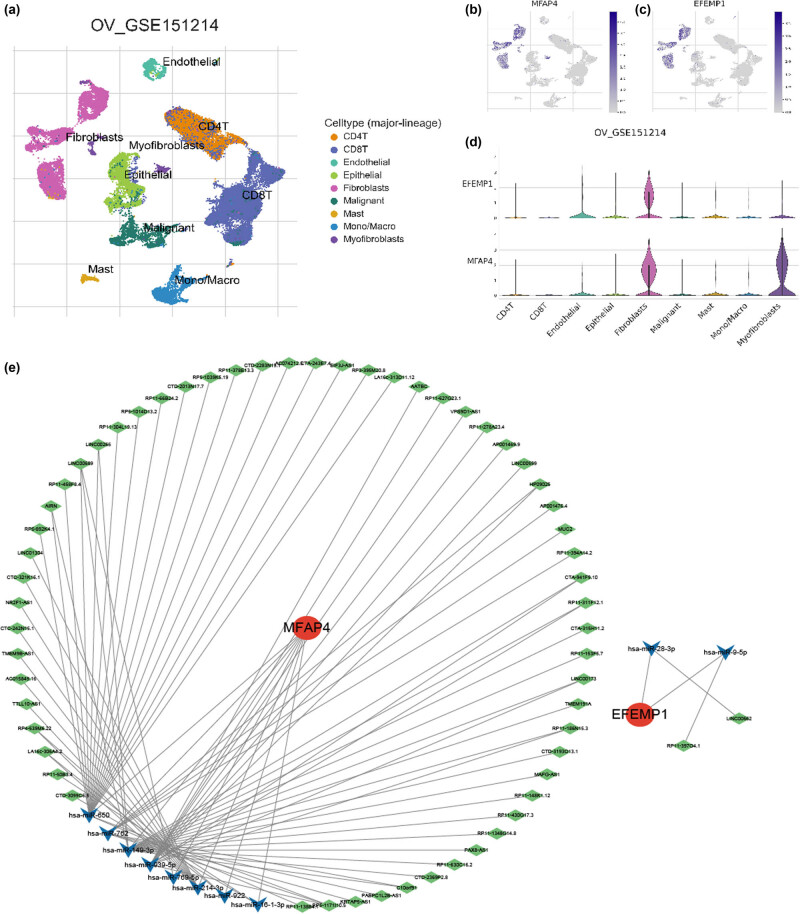
Single cell analysis and ceRNA network construction. (a) UMAP visualization of the GSE151214 dataset, cells are colored by cluster. (b) and (c) Feature plots depicting the expressions of MFAP4 and EFEMP1 in all cell types. (d) Violin plots show the expression level of MFAP4 and EFEMP1 in different cell types. (e) Regulatory network of lncRNA–miRNA–mRNA was visualized using Cytoscape.

The TISCH website was used to analyze the expression patterns of core genes in different TME-associated cells using the scRNA-seq dataset GES151214 (Figure 7a). Notably, fibroblasts showed significant expression levels of MFAP4 and EFEMP1 (Figure 7b–d). We then conducted an extensive search across various databases, including TargetScan, miRanda, miRWalk, and miRDB, to explore the potential interactions between mRNAs and miRNAs. This analysis successfully identified 19 significant pairs of miRNA–mRNA interactions. Additionally, we utilized the SpongeScan analysis tool to construct a comprehensive regulatory network for lncRNA–miRNA interactions and discovered 74 pairs of such interactions. As illustrated in Figure 7e, the results demonstrated that MFAP4 was predominantly targeted by miR-650, miR-762, miR-149-3p, miR-939-5p, miR-769-5p, miR-214-3p, miR-922, and miR-16-1-3p along with their associated lncRNAs. EFEMP1 is regulated by miR-28-3p and miR-9-5p along with their related lncRNAs.

## 4 Discussion

OC is characterized by its insidious nature, with over 70% of cases being diagnosed at an advanced stage [27]. Patients whose OC is confined to the ovaries exhibit a 5-year survival rate exceeding 90% [28]. In recent years, there has been a notable interest among young women with early-stage OC in preserving their fertility. However, platinum-based chemotherapy, a common treatment for OC, may exert cytotoxic effects on ovarian follicles, potentially leading to ovarian failure [29]. Currently, several interventions are routinely employed in clinical practice to preserve fertility, including the administration of gonadotropin-releasing hormone agonists and the cryopreservation of oocytes or ovarian tissue [29,30]. In women who undergo chemotherapy following fertility-sparing surgery (FSS), approximately 65–70% are expected to regain ovarian function, with no observed increase in the incidence of congenital malformations post-pregnancy. It is recommended, however, to delay conception for 6–12 months to mitigate the potential adverse effects of chemotherapeutic agents on oocytes [31]. Furthermore, the implementation of prenatal screening through noninvasive prenatal testing technology, along with long-term monitoring of offspring health, is advised [32]. Thus, a multidisciplinary approach that includes gynecologists, oncologists, and psychologists is essential for the effective implementation of fertility preservation strategies in women diagnosed with OC [33,34].

Presently, FSS offers guidance on prognosis and pregnancy outcomes for women, contingent upon the stage and pathological classification of OC [35]. Nonetheless, the presence of chemotherapy resistance in OC is intricately linked to both oncological and obstetric outcomes. Platinum resistance is a complex and multi-faceted process that involves the interplay of multiple genes and various factors [36]. Numerous studies have also focused on identifying the key genes responsible for platinum resistance in OC [37]. To elucidate the pivotal genes responsible for platinum resistance, we employed a range of bioinformatics techniques encompassing WGCNA, DEGs, and multimachine learning algorithms such as LASSO, SVM-RFE, and RF. Through this comprehensive analysis, we successfully identified MFAP4 and EFEMP1 genes that may contribute to chemotherapy resistance in OC patients. Furthermore, our survival analysis revealed a significant association between the expression levels of MFAP4 and EFEMP1 and unfavorable prognosis in OC. Based on the aforementioned findings, we can inform OC patients about the risks associated with preserving fertility by assessing the expression levels of MFAP4 and EFEMP1.

MFAP4 belongs to a family of fibrinogen-related domain proteins and plays a pivotal role in various pathological conditions involving tissue remodeling, including fibrosis, cardiovascular diseases, aging, and cancer [38,39]. MFAP4 exhibits heterogeneous expression levels in various tissues. Notably, in pancreatic adenocarcinoma, MFAP4 was identified as a carrier of sialyl-Lewis x with significantly higher expression compared to control tissues [40]. Conversely, other studies have reported significantly lower levels of MFAP4 expression in lung adenocarcinoma and breast cancer [41,42]. In our investigation of platinum-resistant OC patients, we observed elevated expression levels of MFAP4, which correlated with poor prognosis. Consistently, an analysis focusing on platinum drug resistance in OC also supports this finding [43]. EFEMP1, also known as fibulin 3, is a crucial extracellular matrix protein that plays a pivotal role in maintaining the structural integrity and stability of the ECM [44,45]. Specifically, EFEMP1 exhibits diverse expression patterns across different tissues and exerts a dual function in cancer progression [46]. In breast cancer, miR-9-mediated down-regulation of EFEMP1 has been implicated in the transformation of normal fibroblasts into cancer-associated fibroblasts [47]. Proteomic profiling analysis has identified EFEMP1 as a metastatic driver and a potential prognostic biomarker for lung metastasis in osteosarcoma patients [48]. Moreover, elevated expression levels of EFEMP1 have been shown to promote HeLa cell proliferation [49], whereas its overexpression has been observed in chemoresistant variants of the A2780 OC cell line [50]. Additionally, upregulation of EFEMP1 has been associated with enhanced invasiveness and metastatic potential in OC through the activation of the AKT signaling pathway [51]. These studies show that these two genes are closely related to cancer progression and drug resistance.

Chemotherapy resistance is a complex process that can be influenced by a variety of factors, including the EMT, TME, and so on [52–54]. EMT is a biological process by which epithelial cells undergo various phenotypic changes along the epithelial–mesenchymal axis. These cellular states exhibit distinct characteristics such as stemness, invasiveness, drug resistance, and metastatic potential, contributing to cancer metastasis and relapse [55,56]. SNAIL1 and SNAIL2, as major inducers of EMT, a study demonstrated that SNAIL1 is implicated in cisplatin resistance of OC cells, no such association was observed for SNAIL2 [57]. Studies have investigated the proteomic disparities between carboplatin-sensitive and carboplatin-resistant OC cells, revealing a heightened expression of EMT modulators, including G6PD, AKR1B1, ITGAV, and TGFβ1, in the resistant cohort [58]. Studies have shown that estrogen suppresses EFEMP1 and inhibits the Wnt/β-catenin signaling pathway to prevent EMT in endometrial carcinoma [59]. The interaction between EFEMP1 and STEAP1 facilitates the initiation of Wnt/β-catenin and TGF-β/Smad2/3 axes, leading to the induction of EMT, thereby promoting the infiltration and migration of osteosarcoma cells [60]. Limited research has been conducted on the correlation between MFAP4 expression and EMT. A single study revealed enrichment of extracellular matrix factors CHRDL1 and MFAP4 secreted by adult chondrocytes, along with enhanced networks involved in cartilage development pathways and EMT [61]. In the present study, we found that MFAP4 and EFEMP1 were positively correlated with EMT, indicating that these genes may contribute to chemoresistance.

The TME is also an important factor in chemotherapy resistance. The TME is composed of a variety of cells, including cancer cells, immune cells, and fibroblasts, as well as extracellular matrix proteins and other molecules, which are characterized by hypoxia, interstitial high pressure, and inflammatory reactivity and are closely related to tumor growth, metastasis, and drug resistance [62]. The high expression of MFAP4 and EFEMP1 exhibited increased stromal, immune, and ESTIMATE scores; however, they demonstrated an inverse correlation with tumor purity. CD8^+^ T cells have the ability to selectively eliminate tumor cells; however, the phenomenon of “tumor and T cell coexistence” indicates that CD8^+^ T cells are dysfunctional during tumorigenesis [63]. MFAP4 was found to be positively correlated with CD8^+^ T cells in a study of endometriosis [64]. In this study, we found that MFAP4 positively correlated with CD8^+^ T cells. Tumor-associated macrophages are important regulatory cells involved in tumor-associated inflammation, and the subtypes that promote tumor growth in the TME are mainly the polarized type (M2-TAM) [65]. Our results demonstrate that EFEMP1 is positively correlated with M2-TAM and MFAP4 is closely correlated with M2-TAM. The above studies show that these two genes are related to the remodeling of the TME.

Despite the favorable outcomes derived from the bioinformatic investigation of OC, this study has certain limitations. First, the preliminary results appear promising, but it is imperative to conduct extensive experiments and perform comprehensive data analysis to ascertain the universality and reliability of these findings. Second, MFAP4 and EFEMP1 have been well studied in various cancers, and the intricate mechanisms underlying the upregulation of MFAP4 and EFEMP1 in platinum-resistant OC patients remain unclear, necessitating further exploration in future research.

## 5 Conclusion

In conclusion, we identified MFAP4 and EFEMP1 as potential biomarkers that could be used to predict the response to platinum-based chemotherapy and determine survival outcomes in patients with OC. MFAP4 and EFEMP1 are closely related to EMT and the TME, which is consistent with the mainstream studies on platinum resistance. Risk stratification can be achieved through testing these markers, thereby providing comprehensive information to patients considering fertility preservation. Understanding these factors and how they contribute to chemotherapy resistance is crucial for developing more effective treatment strategies and improving the outcomes of patients with OC.
